# A Subcellular Quantitative Proteomic Analysis of Herpes Simplex Virus Type 1-Infected HEK 293T Cells

**DOI:** 10.3390/molecules24234215

**Published:** 2019-11-20

**Authors:** Weiwei Wan, Liangjie Wang, Xi Chen, Shenglin Zhu, Weijuan Shang, Gengfu Xiao, Lei-Ke Zhang

**Affiliations:** 1 State Key Laboratory of Virology, Wuhan Institute of Virology, Chinese Academy of Sciences, Wuhan 430071, China; weiwei.wan.cas.whiov@gmail.com (W.W.); cqubiozsl@gmail.com (S.Z.); shangweijuan@wh.iov.cn (W.S.); 2 University of the Chinese Academy of Sciences, Beijing 100049, China; 3 Hubei Key Laboratory of Purification and Application of Plant Anti-Cancer Active Ingredients, School of Chemistry and Life Sciences, Hubei University of Education, Wuhan 430205, China; wangliangjie@hue.edu.cn; 4 Department of Biological Mass Spectrometry, Wuhan Institute of Biotechnology, Wuhan 430074, China; chenxi@spec-ally.com; 5 Medical Research Institute, Wuhan University, Wuhan 430074, China

**Keywords:** quantitative proteomics, herpes simplex virus type 1, virus–host interaction, IFITM3, CHCHD2, TRIM27

## Abstract

Herpes simplex virus type 1 (HSV-1) is widespread double-stranded DNA (dsDNA) virus that establishes life-long latency and causes diverse severe symptoms. The mechanisms of HSV-1 infection and HSV-1’s interactions with various host cells have been studied and reviewed extensively. Type I interferons were secreted by host cells upon HSV infection and play a vital role in controlling virus proliferation. A few studies, however, have focused on HSV-1 infection without the presence of interferon (IFN) signaling. In this study, HEK 293T cells with low toll-like receptor (TLR) and stimulator of interferon genes protein (STING) expression were infected with HSV-1 and subjected to a quantitative proteomic analysis. By using a subcellular fractionation strategy and high-performance mass spectrometry, a total of 6607 host proteins were quantified, of which 498 proteins were differentially regulated. A bioinformatics analysis indicated that multiple signaling pathways might be involved in HSV-1 infection. A further functional study indicated the role of Interferon-induced transmembrane protein 3 (IFITM3), Coiled-coil-helix-coiled-coil-helix domain-containing protein 2 (CHCHD2), and Tripartite motif-containing protein 27 (TRIM27) in inhibiting viral DNA replication and proliferation. Our data provide a global view of host responses to HSV-1 infection in HEK 293T cells and identify the proteins involved in the HSV-1 infection process.

## 1. Introduction

Herpes simplex virus type 1 (HSV-1), a member of the alpha-herpesvirus family, is a contagious human pathogen. According to a WHO report [1], HSV-1 is estimated to have infected 67% of the world’s population under the age of fifty. HSV-1 infection establishes life-long latency with reactivation triggered by stimuli like UV light, tissue damage, or compromised immunity [2]. The severity of HSV-1 infection varies from mild symptoms, such as cold sores and watery blisters on the skin or mucous membranes, to life-threatening encephalitis in humans [3]. 

As a double-stranded DNA virus, HSV-1 has a linear genome of 152 kbp surrounded by a protein layer called a tegument and a bilayer envelope with viral glycoproteins [4]. During infection, the virus first binds the receptors present on host cell surfaces through glycoproteins of the viral envelope and then transitions from attachment to penetration, leading to the release of the viral capsid and tegument proteins into the cytoplasm. Later, viral capsids with tegument proteins use the microtubule network to transfer to the nuclear envelope, where they interact with nuclear pores and release their uncoated genomes into the nucleoplasm to perform viral transcription and replication. During these processes, the viruses will utilize various host cell systems to promote their own replication.

As a powerful tool with high throughput and accuracy, MS-based proteomic analyses have been used increasingly in the analysis of virus–host interactions, aiming to discover new drug targets or unveil unknown mechanisms. Until now, proteomic analyses of HSV-1 have focused on virally induced changes in the cellular proteome [5,6,7] or protein post-translational modifications [8] and virus–protein interactions [9,10,11] during infection. In this study, a stable isotope-labeled amino acid culture (SILAC)-based quantitative proteomic analysis of HSV-1 infected HEK 293T cells was performed, aiming to explore the effects of HSV-1 infection on the host proteome. An RNAi-based functional analysis was employed to explore the role of several regulated host proteins in viral replication. In addition, the host cell activates various antiviral responses to limit viral infection. The entry of HSV-1 into host cells triggers a series of both innate and adaptive immune responses, in which innate immunity plays a central role in determining the final fate of infection [12]. HSV-1 and Sendai virus (SeV) have been employed as models for DNA and RNA viruses, respectively, and have been extensively used in virus–host interaction studies, especially in studies investigating the mechanisms of the host’s immune response to DNA viruses and RNA viruses. A comparative analysis of the effects of SeV and HSV-1 infection on the host proteome has never been reported. Our previous SILAC-based quantitative analysis of SeV-infected HEK 293T cells highlighted several biological processes that may be involved in the viral replication process and virus-induced innate immune response [13]. In this study, we also compared the effects of SeV infection and HSV-1 infection on HEK 293T cells, and the biological processes regulated by both HSV-1 and SeV were identified. Our quantitative proteomic analysis of HSV-1- and SeV-infected cells can also help us to better understand HSV-1- or SeV-induced immune responses. 

## 2. Results

### 2.1. HSV-1 Replicates Effectively in HEK 293T Cells without Inducing IFN-β Production

**Figure 1:**
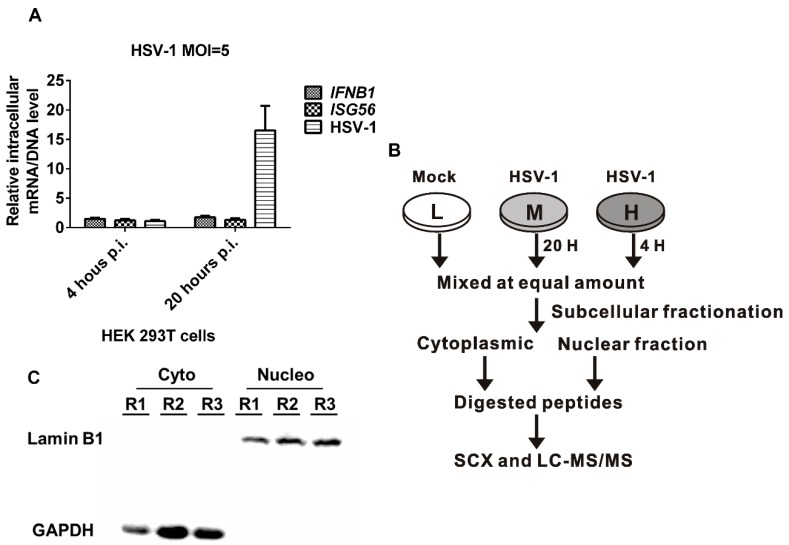
Subcellular quantitative proteomic analysis of herpes simplex virus type 1 (HSV-1)-infected HEK 293T cells. (**A**) Intracellular levels of HSV-1 genome DNA and *IFNB1* and *ISG56* mRNAs in HSV-1 infected HEK 293T cells. Mock- or HSV-1 (MOI = 5)-infected HEK 293T cells were harvested at 4 h p.i. and 20 h p.i. Total RNA was extracted and reverse transcribed into the cDNA to quantify the intracellular mRNA level of *IFNB1* and *ISG56* using quantitative RT-PCR. The total DNA was extracted, and the intracellular DNA level of the HSV-1 genome was measured with quantitative RT-PCR. (**B**) The MS analysis workflow of the stable isotope-labeled amino acid culture (SILAC). (**C**) Confirming the subcellular fractionation efficiency by Western blot. Cytoplasmic and nuclear fractions from the three biological replicates (R1, R2, and R3) were subjected to Western blot analyses. Glyceraldehyde-3-phosphate dehydrogenase (GAPDH) and lamin B1 were used as markers of cytoplasmic and nuclear proteins, respectively. H: hours.

To explore the effects of HSV-1 infection on the proteome of HEK 293T cells, we performed a SILAC-based quantitative proteomic analysis of HSV-1-infected HEK 293T cells at 4 and 20 h post infection (p.i.), which represent the early and late phases of HSV-1 infection, respectively. HEK 293T cells are highly permissive to HSV-1 infection and have low TLR and STING expression, and thus their innate immune response during infection with a DNA virus is weak, resulting in weak activation of Interferon-stimulated genes (ISGs) and related biological processes. We performed a quantitative proteomic analysis on HEK 293T cells to minimize the effect of interferons on the regulation of host proteins and biological processes. Twenty hours p.i. was chosen as the time point for late phase, because we found that the HSV-1 DNA level was much higher (Figure 1A), and no significant apoptosis can be observed at this time point. This observation is consistent with the understanding that HSV-1 takes around 16–20 h to complete a lytic cycle [5]. However, the intracellular mRNA levels of *IFNB1* and *ISG56* were comparable between 4 and 20 h p.i., suggesting that type I IFN (IFN-I) production was not activated in HSV-1-infected HEK 293T cells, which is consistent with the literature [14,15]. 

### 2.2. SILAC-Based Quantitative Proteomic Analysis of HSV-1 Infected HEK 293T Cells 

To perform a quantitative proteomic analysis of HSV-1-infected host cells, HEK 293T cells were differentially labeled by being cultured in triple-SILAC media for seven passes, and then infected with HSV-1 at a multiplicity of infection (MOI) of 5 or mock treated (Figure 1B). Then, the infected cells were harvested at 4 h p.i. and 20 h p.i., and the mock cells were collected and mixed in an equal amount. The cells were then subcellularly fractionated into cytoplasmic and nuclear fractions, and the subcellular fractionation efficiency was confirmed by detecting lamin B1 and glyceraldehyde-3-phosphate dehydrogenase (GAPDH) using Western blot (Figure 1C). Then, the fractionated proteins were digested into peptides and further fractionated with strong cation exchange chromatography (SCX) for MS analysis. 

**Figure 2:**
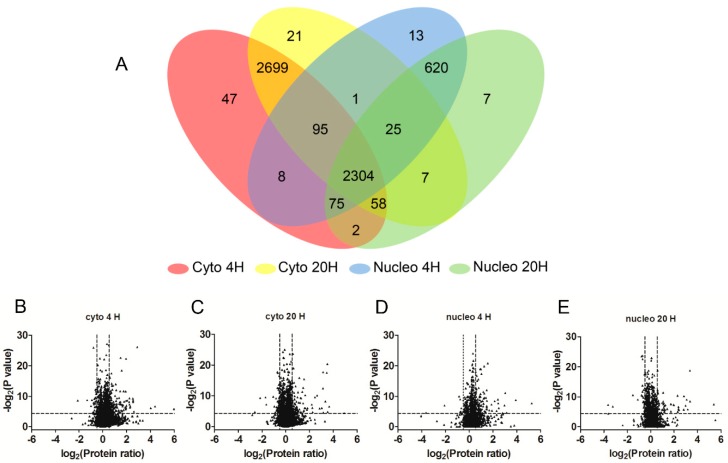
Overview of proteins quantified by SILAC-MS. (**A**) A Venn diagram of the quantified host proteins. A total of 6607 host proteins were quantified, among which 5784 were from the cytoplasmic fraction (cyto) and 3382 were from the nuclear fraction (nucleo). In the cytoplasmic fraction, 5516 and 5661 proteins were quantified at 4 h p.i. and 20 h p.i., respectively. In the nuclear fraction, 3225 proteins were quantified at 4 h p.i. and 3266 proteins were quantified at 20 h p.i. H: hours. (**B**–**E**) Volcano plots of the differentially expressed proteins between the HSV-1- and mock-infected HEK 293T cells.

Three independent biological replicates were subjected to an MS analysis, and ProteinPilot v 5.0 was used for peptide identification and quantitation. Peptides with a confidence score above 95% and a ratio >0 and <99 were considered as being quantified. Proteins with at least one quantified peptide were included for further analyses. Using these criteria, a total of 6607 host proteins were quantified [false discovery rate (FDR) < 1%)], among which 5784 were from the cytoplasmic fraction and 3382 were from the nuclear fraction (Figure 2A). In the cytoplasmic fraction, the numbers of quantified proteins at 4 h p.i. and at 20 h p.i. were 5516 and 5661, respectively. In the nuclear fraction, 3225 quantified proteins were from 4 h p.i. and 3266 quantified proteins were from 20 h p.i. (Figure 2A).

The average values of the protein ratio from the three biological replicates and the *p*-value were calculated and plotted on a volcano plot (Figure 2B–E). Regulated proteins were divided into three categories: category A (high confidence), category B (medium confidence), and category C (low confidence). Based on these criteria, 498 proteins were differentially regulated with high confidence; 483 proteins were differentially regulated with medium confidence; and 923 proteins were differentially regulated with low confidence (Table S1). 

### 2.3. Validation of SILAC-MS Data by Western Blot

**Figure 3:**
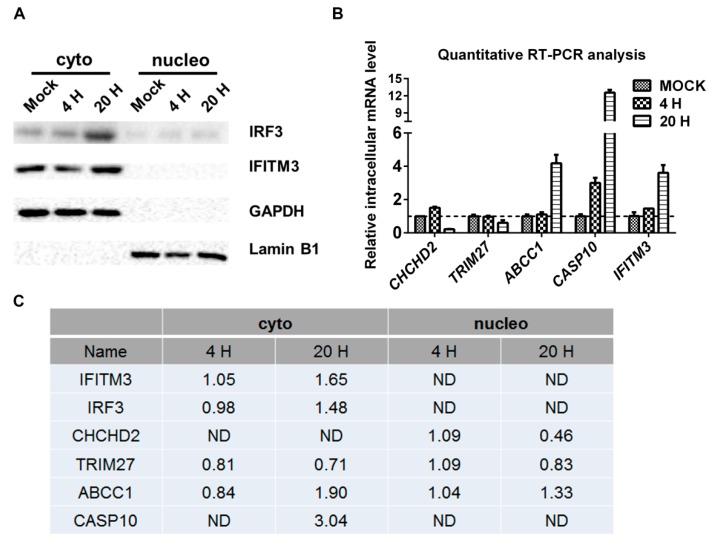
Validation of the protein regulation data by quantitative RT-PCR and Western blots. (**A**) Western blot analysis of the host proteins. Proteins from the cytoplasmic and nuclear fractions of the mock- or HSV-1-infected (MOI = 5) HEK 293T cells were extracted for Western blot analysis. Lamin B1 and GAPDH were used as internal controls for nuclear (nucleo) and cytoplasmic (cyto) proteins, respectively. (**B**) Quantitative RT-PCR analysis of the intracellular mRNA level of selected proteins. Mock- or HSV-1-infected (MOI = 5) HEK 293T cells were harvested, and the total mRNA was extracted and reverse transcribed into cDNA for quantitative RT-PCR analysis. The values are presented as the mean ± SD of three replicates. (**C**) SILAC-MS data for selected proteins. ND: not detected; H: hours.

We then performed Western blots on two differentially regulated proteins: Interferon regulatory factor 3 (IRF3) (category B) and IFITM3 (category C). As shown in Figure 3A, the Western blots indicated that IRF3 and IFITM3 were upregulated at 20 h p.i., but hardly at 4 h p.i. in the cytoplasm. The SILAC-MS data indicated that IRF3 and IFITM3 were not regulated at 4 h p.i. in the cytoplasm, but upregulated at 20 h p.i. Both the Western blots and SILAC-MS analysis did not detect IRF3 and IFITM3 in the nucleus. Although the confidence of regulation information of IRF3 and IFITM3 was low, our Western blot results confirmed their regulations. 

A quantitative RT-PCR analysis was performed on five regulated proteins. As demonstrated in Figure 3B, the intracellular mRNA level of *CHCHD2* was downregulated at 20 h p.i. but hardly at 4 h p.i., while the intracellular mRNA levels of multidrug resistance-associated protein 1 (*ABCC1)*, Caspase-10 (*CASP10)*, and *IFITM3* were upregulated at 20 h p.i., and the intracellular mRNA level *CASP10* also increased at 4 h p.i. These regulations were consistent with the SILAC-MS data (Figure 3C), suggesting that these proteins may be regulated at the transcriptional level. TRIM27 was identified by the MS data as being downregulated, while the intracellular mRNA level of *TRIM27* did not change significantly, suggesting that the downregulation of TRIM27 occurred at the protein level, which agreed with a recent study and indicated that TRIM27 is a degradation target of HSV-1 ICP0 [11]. 

### 2.4. Gene Ontology (GO) Analysis Reveals Biological Processes Regulated by HSV-1 Infection

**Figure 4:**
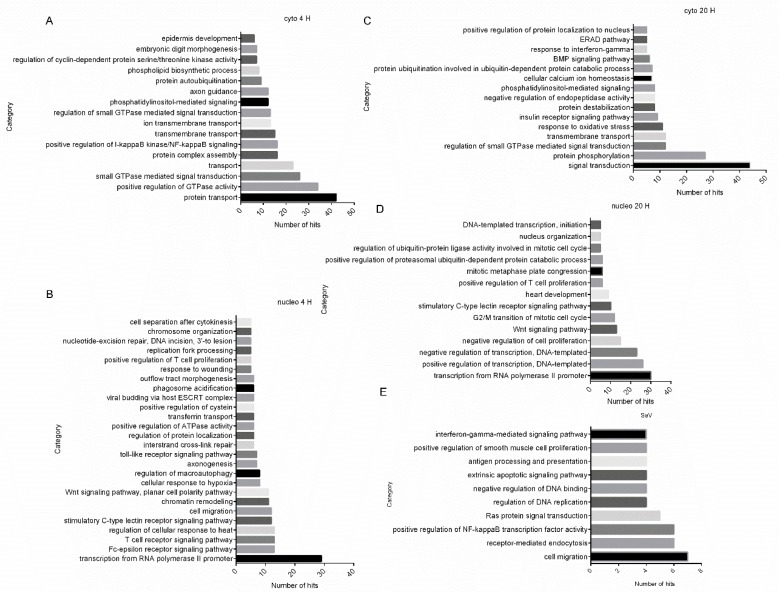
Gene ontology analysis of differentially regulated proteins in HSV-1-infected HEK 293T cells. Regulated proteins identified at 4 h p.i. (**A**) or 20 h p.i. (**C**) in cytoplasmic (cyto) or regulated proteins identified at 4 h p.i. (**B**) or 20 h p.i. (**D**) in nuclear (nucleo) fractions were submitted to the Database for Annotation, Visualization, and Integrated Discovery (DAVID) website for gene ontology (GO) analysis, respectively. All proteins quantified in this study were set as the background. H: hours. (**E**) Regulated proteins in both SeV- and HSV-1-infected cells were submitted to DAVID to perform a GO analysis based on biological processes.

The differentially regulated proteins in both fractions at both time points were submitted to the Database for Annotation, Visualization, and Integrated Discovery (DAVID) to perform a GO analysis based on “biological process” separately, with all the proteins quantified in all the fractions as the background (Table S2). As shown in Figure 4A–D, the categories “positive regulation of I-kappaB kinase/NF-kappaB signaling”, “positive regulation of GTPase activity”, “protein transport”, and “ion transmembrane transport” were over-represented by the regulated proteins at 4 h p.i. in the cytoplasmic fraction, while the categories “transcription from RNA polymerase II promoter”, “viral budding via host endosomal sorting complexes required for transport (ESCRT) complex”, “toll-like receptor signaling pathway”, and “Wnt signaling pathway” were over-represented by the regulated proteins at 4 h p.i. in the nuclear fraction. The categories “signal transduction”, “ion trans-membrane transport”, “response to oxidative stress”, and “response to interferon-gamma” were over-represented by the regulated proteins at 20 h p.i. in the cytoplasmic fraction, while “transcription from RNA polymerase II promoter”, “Wnt signaling pathway”, and “positive regulation of T-cell proliferation” were over-represented by the regulated proteins at 20 h p.i. in the nuclear fraction. The over-represented categories suggest that these biological processes are more vulnerable under HSV-1 infection than other pathways. More regulated biological processes were identified in the nuclear fraction, possibly because the HSV-1 genome mainly replicates in the nucleus of infected cells.

We also compared our data with previous quantitative proteomic analyses of SeV-infected HEK293T cells and found that 75 regulated proteins were common. A GO analysis based on biological processes indicated that these proteins were enriched in the following categories: “cell migration”, “receptor-mediated endocytosis”, and “positive regulation of NF-kappaB transcription factor activity” (Figure 4E), suggesting that these biological processes can be regulated by both HSV-1 and SeV infections. 

### 2.5. Roles of IFITM3, CHCHD2, and TRIM27 on Virus Proliferation

**Figure 5:**
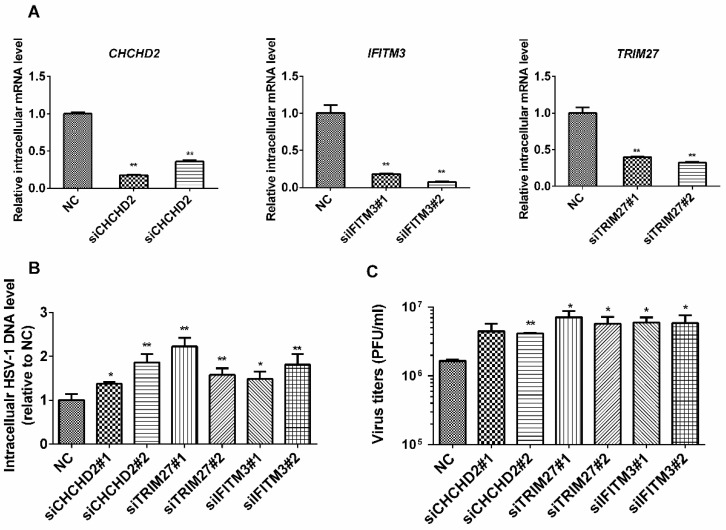
Functional analysis of regulated proteins on virus proliferation. (**A**) The knockdown efficiency of siRNAs on targeted proteins in HEK 293T cells. HEK 293T cells were transfected with the indicated siRNAs, and at 24 h post transfection, the total mRNA was extracted and transcribed into cDNA for quantitative RT-PCR analysis. Cells transfected with scrambled siRNAs were used as the negative control (NC). (**B**) The effects of the knockdown of selected proteins on virus DNA levels. HEK 293T cells were transfected with mock or siRNAs for selected genes, followed by a 24 h culture and then infected with HSV-1 at an MOI of 5. Total DNA was harvested, and the intracellular level of the HSV-1 DNA was measured with quantitative RT-PCR analysis. Cells transfected with scrambled siRNAs are indicated as NC. Y-axis: The intracellular level of HSV-1 DNA relative to NC. (**C**) The effects of the knockdown of selected proteins on virus titer in the supernatant. HEK 293T cells were transfected with mock or siRNAs for the selected genes, followed by a 24 h culture and then infected with HSV-1 at an MOI of 5. Supernatants were collected and viral titers in the supernatants was measured. The values are presented as the mean ± SD of three replicates. The cells transfected with scrambled siRNAs are indicated as NC. *, *p* < 0.05; **, *p* < 0.01.

To further explore the significance of our data, an interference RNA (RNAi)-based functional study on selected regulated proteins was performed. These proteins were selected based on a comparison with our previous quantitative analysis of RNA virus-infected host cells. CHCHD2 was also regulated by SeV [13], and IFITM3 was regulated by JEV [16], while TRIM27 was only regulated by HSV-1. HEK 293T cells were transfected with siRNAs against *IFITM3*, *CHCHD2*, and *TRIM27*, and 24 h later, the cells were collected, and intracellular mRNAs were harvested for quantitative RT-PCR to test the knockdown efficiency of these siRNAs (Figure 5A). All six pairs of siRNAs decreased gene expression significantly and, therefore, were used for the following assays. Next, HEK 293T cells were transfected with the indicated siRNAs, and 24 h later, the cells were infected with HSV-1 at an MOI of 5. At 20 h p.i., both the cells and supernatants were collected, and the intracellular viral genomic DNA level was measured by quantitative RT-PCR. As shown in Figure 5B, the intracellular levels of HSV-1 DNA were significantly higher in cells transfected with siRNAs against *IFITM3*, *CHCHD2*, or *TRIM27*, suggesting that the knockdown of *IFITM3*, *CHCHD2*, or *TRIM27* increased the intracellular level of HSV-1 DNA. We also determined viral titers in the supernatants, and found that the knockdown of *IFITM3*, *CHCHD2*, or *TRIM27* also increased the HSV-1 titer (Figure 5C). This implies that IFITM3, CHCHD2, and TRIM27 play positive roles in defending against HSV-1 infection.

## 3. Discussion

HSV-1 is a widespread contagious pathogen and has been studied for decades. During the last few decades, MS-based proteomic methods have contributed significantly to revealing more factors and mechanisms involved in HSV-1 infection and the corresponding host responses. A quantitative proteomic analysis using a 2D gel strategy was performed to explore the effects of HSV-1 infection on the host proteome [5,6,17]. However, due to the limits of the gel-based strategy, the number of regulated proteins identified was low. Berard et al. also performed a SILAC-based quantitative proteomic analysis of HSV-1 infected cells [7], but they only used two replicates. In this study, a comprehensive analysis of HSV-1-infected HEK 239T cells was performed. To reduce the complexity of the proteins analyzed, cells were sub-fractionated into nuclear and cytoplasmic fractions, and three replicates were performed. A total of 6607 host proteins were quantified, of which 498 proteins were differentially regulated (Table S1). A GO analysis based on a biological process highlighted several proteins groups that were affected by HSV-1 infection. The significance of the NF-κB pathway [18] and the ubiquitin–proteasome system [19] on virus–host interactions during HSV infection have been reported repeatedly. In agreement with these reports, “NIK/NF-κB signaling” and “protein ubiquitination involved in ubiquitin-dependent protein catabolic process” were enriched in our GO data. The “positive regulation of GTPase activity”, “negative regulation of transcription from RNA polymerase II promoter”, and “negative regulation of cell proliferation” enriched in this study coincided with previous results that showed HSV can activate dynamin 2 GTPase [20] and alter the loading and positioning of RNA Polymerase II on host genes [21] and that the translation initiation factor eIF2α is inhibited by protein kinase RNA-activated (PKR) activation [22], respectively. 

IFN-inducible transmembrane (IFITM) protein 3 was a member of the first identified ISGs. A wealth of data has suggested that IFITM3 strongly protects host cells against a broad range of viruses, including SARS coronavirus, influenza A virus, human immunodeficiency virus type 1, and Zika virus [23]. The antiviral effects of IFITM3 might be attributed to the blockage of virus entry by inhibiting the fusion of the virus membrane, though the mechanisms are still unclear. One possible mechanism is that by binding with vesicle-associated membrane protein-associated protein A (VAPA), IFITM3 disrupts intracellular cholesterol homeostasis and blocks viral release to cytosol [24]. This is consistent with our data and explains our finding that HSV-1 increases IFITM3 expression, and knockdown of this gene leads to higher virus proliferation.

The role of the coiled-coil-helix-coiled-coil-helix domain containing 2 (CHCHD2) during herpesvirus infection has not been reported yet. CHCHD2 is a mitochondrial protein involved in apoptosis pathways by binding to BCL-xL and in stabilizing cytochrome c in the respiratory complex [25]. Its mutation has been linked to Parkinson’s disease [26]. Song et al. reported CHCHD2 expression in liver cancer and suggested that it might be a biomarker of hepatocellular carcinoma [27]. Our previous study found that CHCHD2 can be induced by Zika virus infection in HeLa cells and may promote ZIKV replication and inhibit virus-induced IFN-I production [28]. We found that CHCHD2 was downregulated by HSV-1 infection in HEK 293T cells, and the knockdown of CHCHD2 can increase HSV-1 production in a cell culture supernatant (Figure 5). However, the detailed role of CHCHD2 on virus proliferation at the molecular level is still unknown, and more studies are needed.

In sum, by using a quantitative proteomic analysis of HSV-1-infected HEK 293T cells at two time points, we provided a global view of host–viral protein interactions during infection in the absence of type I IFN. We identified multiple pathways that may participate in HSV-1 infection through a GO analysis. A functional assay suggested a positive role of three proteins: IFITM3, CHCHD2, and TRIM27 in the anti-HSV-1 process. The role of CHCHD2 in inhibiting HSV-1 replication has never been reported. Further studies on these proteins are necessary for further understanding of the underlying mechanisms and may help us discover new targets for antiviral treatments.

## 4. Materials and Methods

### 4.1. Cell and Virus

HEK 293T cells obtained from CCTCC (China Center for Type Culture Collection, Wuhan, China) were grown in Dulbecco’s modified Eagle’s medium (DMEM, GIBCO, Grand Island, NE, USA) supplemented with 1% penicillin/streptomycin (GIBCO) and 10% fetal bovine serum (FBS, GIBCO) at 37 °C with 5% CO_2_. The HSV-1 strain 129 (H129) was used in this study, and virus was stocked at −80 °C.

For SILAC labeling, HEK 293T cells were cultured in a SILAC medium (Thermo, Waltham, MA, USA) containing 10% dialyzed FBS (Thermo) and l-arginine and l-lysine (R0K0, “light”), l-arginine-13C6 and l-lysine-4,4,5,5-d4 (L) (R6K4, “medium”), or l-arginine-13C6, 15N4 and l-lysine-13C6, 15N2 (R10K8, “heavy”) (Sigma, St. Louis, MO, USA). Cells were cultured in the SILAC medium for at least seven doublings to ensure that the labeled amino acids were incorporated into cellular proteins. Cells cultured in different mediums were infected with HSV-1 at an MOI of 5 or mock treated, and harvested at 4 or 20 h p.i. Then, mock-treated cells (light), HSV-1 infected cells harvested at 4 h p.i. (heavy), and HSV-1 infected cells harvested at 20 h p.i. (medium) were mixed in an equal amount.

### 4.2. Subcellular Fractionation

Subcellular fractionation was performed with a nuclear and cytoplasmic protein extraction kit (Beyotime, Shanghai, China). The cell pellets were first washed three times with pre-chilled PBS and then resuspended in pre-chilled nuclear and cytoplasmic protein extraction kit buffer A and incubated on ice for 15 min. Then, nuclear and cytoplasmic protein extraction kit buffer B was added, and the mixture was vortexed three times and centrifuged at 500 × *g* for 5 min. The supernatant was collected as the cytoplasmic fraction. The pellet was resuspended in nuclear and cytoplasmic protein extraction kit buffer C, incubated on ice for 15 min, and centrifuged at 13,200 × *g* for 15 min. The supernatant was then collected as the nuclear fraction. 

### 4.3. Protein Extraction and Digestion

Extracted proteins were collected and the protein concentration was measured with a BCA assay. Extracted proteins were reduced with 10 mM DTT at 56 °C for 30 min, alkylated with 40 mM IAA in the dark for 30 min, and left at room temperature for 1 h. The proteins were digested with trypsin (Promega, Madison, WI, USA) at a ratio of 1:50 (trypsin/protein *w*/*w*) at 37 °C overnight. Digested peptides were desalted with a SepPak C18 cartridge (Waters, Milford, CT, USA) and dried by SpeedVac (Thermo). Then, the digested peptides were fractionated with SCX, and the fractionated peptides were desalted with Ziptip (Millipore, Billerica, MA, USA) and stored at −80 °C before LC-MS/MS analysis.

### 4.4. Liquid Chromatography/Mass Spectroscopy (LC-MS/MS) Analysis

To quantify the differentially expressed proteins in the samples, an LC-MS/MS analysis was performed as described previously [29]. A hybrid quadrupole-TOF LC/MS/MS mass spectrometer (TripleTOF 5600+, AB SCIEX) was coupled to a nanospray ion source. Peptides were first loaded onto a 5 µm-C18 trap column (5 × 0.3 mm; Agilent Technologies, Santa Clara, CA, USA) and then separated on a C18 analytical column (75 μm × 150 mm, 3 μm particle size, 100 Å pore size; Eksigent, Dublin, OH, USA). A 100 min gradient was established using mobile phase A (3% DMSO, 96.9% H2O, and 0.1% formic acid) and mobile phase B (3% DMSO, 96.9% ACN, and 0.1% formic acid), with a constant flow rate of 300 nL/min. For the MS/MS analysis, each scan cycle consisted of one full-scan mass spectrum (with the *m*/*z* ranging from 350 to 1500 and charge states from 2 to 5) followed by 20 MS/MS events. The threshold count was set to 120 to activate MS/MS accumulation, and the former target ion exclusion was set at 18 s. The tandem mass spectra were extracted by Peakview version 2.0 (AB SCIEX, Framingham, MA, USA). The mass spectrometry proteomics data were deposited to the ProteomeXchange Consortium via the PRIDE [30] partner repository with the dataset identifier PXD015887.

### 4.5. Protein Identification and Quantification

MS spectra were submitted to ProteinPilot version 5.0.1 (AB SCIEX) to perform peptide identification and quantification. The UniProt_Human database (download at 201701) containing HSV-1 proteins was used. The search parameters were as follows: sample type: SILAC (Lys+4D, Arg+6; Lys+8, Arg+10); cysteine alkylation: iodoacetamide; digestion: trypsin; instrument: tripleTOF 5600; miss cleavages tolerance: 2; fixed modification: carbamidomethyl Cys; variable modification: none; MS1 initial mass error tolerance value: 0.05 Dalton; MS2 initial mass error tolerance value: 0.1 Dalton. The false discovery rate (FDR) analysis in ProteinPilot uses a “decoy database searching” strategy, and the FDRs of the ProteinPilot search results were all set as lower than 1% of the protein level.

For protein quantification, all quantified peptides were exported, and only peptides with a confidence score >95% were kept for further analysis. In each replicate, the protein ratio was calculated by the weighted averaging ratios of its peptides, with peptide intensity as the weight. The protein ratio values used for the bioinformatic analysis were the weighted averages of the three biological replicates, while the *P*-value for the protein ratio was assessed using a student’s t-test (Table S1). All the quantified proteins that were presented contained at least one quantified peptide. One peptide quantitative information, as long as the peptide is unique to the protein, was also used as the quantitative information of the protein. The cut-off for differentially regulated proteins was set as described in the previous study [31]. Briefly, the Gaussian distribution of the protein ratios was analyzed, and the values deviating from the mean of the normally distributed data by 1.96 standard deviations (SD) were considered as the cut-off values. Regulated proteins were divided into three categories: category A: proteins with ratios greater than the upregulated or lesser than the downregulated cut-off values and a *p*-value for a protein ratio of <0.05, and these proteins are high confidence regulated proteins; category B: proteins with ratios greater than the upregulated or lesser than the downregulated cut-off values in at least two independent replicates; category C: for proteins only quantified in one replicate, the ratios should be greater than the upregulated or lesser than the downregulated cut-off values, and these are low confidence regulated proteins.

### 4.6. Gene Ontology Analysis

To perform the GO analysis, differentially regulated proteins were submitted to DAVID [32], with all the quantified proteins in this study set as the background. Proteins were classified into different categories based on their roles in biological processes, and a statistical overrepresentation test was performed. P-values were assessed with a binomial test and corrected for multiple tests using a Bonferroni procedure. Only those categories with a *p*-value of 0.05 were considered over- or under-represented.

### 4.7. Cell Transfection

For siRNA transfection, an siRNA was added in Opti-MEM and then mixed with Opti-MEM containing RNAiMAX (Invitrogen). The mixture was incubated at room temperature for 5 min, and 50 μL mixed solution was added to pre-seeded HEK 293T cells in 24-well plates per well. At 24 h post transfection, the cells were infected with HSV-1. At 20 h p.i., both the cell and supernatant were collected, and the viral genome and titer were measured. The final concentration of the siRNA used was 40 nM. The siRNA sequences (5’–3’) were as follows: TRIM27#1-GAAUUAAGAGAGGCUCAGUUA; TRIM27#2-GCCCUACUUCAGUCUGAGUUA; IFITM3#1-UCGUCAUCCCAGUGCUGAU; IFITM3#2-CCCACGUACUCCAACUUCC; CHCHD2#1-ACAGAGCUUGAUGUCACCCUG; CHCHD2#2-CAGUGGAGGAAGUAAUGCUGA.

### 4.8. Western Blot Analysis

Cell samples were lysed with a radio immunoprecipitation assay (RIPA) lysis buffer (Beyotime, China) and subjected to SDS-PAGE. Proteins in the gel were transferred onto a polyvinylidene fluoride (PVDF) membrane (Millipore) and blocked with 5% non-fat milk in TBST buffer. The blots on the membrane were detected by primary antibodies and corresponding horseradish peroxidase-conjugated secondary antibodies (ProteinTech, Wuhan, China). These antibodies were diluted in a TBST buffer containing 5% non-fat milk, and the membrane was washed with a TBST buffer containing 0.1% Tween-20. Finally, the membrane was visualized by enhanced chemiluminescence (ECL) (Millipore), and protein bands were visualized by image using the ChemDoc MP Imaging System (Bio-Rad, Hercules, CA, USA). A mouse monoclonal antibody against GAPDH (ABclonal, Wuhan, China), a rabbit monoclonal antibody against IFITM3 (CST, Danvers, MA, USA), and a rabbit polyclonal antibody against IRF3 (Proteintech) and Lamin B1 (Proteintech) were purchased from indicated companies.

### 4.9. Viral DNA Purification and Quantitationn

Viral DNA was purified with the TIANamp Genomic DNA Kit (Tiangen, Beijing, China). HSV-1-infected HEK 293T cells were collected and lysed with Buffer A from the TIANamp Genomic DNA Kit, and then the DNA was purified following the manufacturer’s protocol. The relative viral DNA level was measured with quantitative RT-PCR. SYBR Premix Ex Taq™ (Applied Biosystems, Foster City, CA, USA) was used as the fluorescent dye on Applied Biosystems 7500 Real-Time PCR Systems. The primers used were synthesized by Sangon (Shanghai, China) and the sequences were as follows (5’–3’): HSV-1, Forward: CGGCCGTGTGACACTATCG, Reverse: CTCGTAAAATGGCCCCTCC; GAPDH, Forward: GAAGGTGAAGGTCGGAGTC, Reverse: GAAGATGGTGATGGGATTTC. The concentration of the primers used for RT-PCR was 400 nM.

### 4.10. Intracellular RNA Extraction and Quantitation

After removal of the supernatant and washing with PBS, the cell samples were lysed with a Trizol reagent (Promega) and total RNA was extracted. The residual genomic DNA was removed by DNAase, and total RNA was reverse transcribed to cDNA using M-MLV reverse transcriptase (Promega). The relative mRNA expression was quantified by quantitative RT-PCR. SYBR Premix Ex Taq™ (Applied Biosystems) was used as the fluorescent dye on Applied Biosystems 7500 Real-Time PCR Systems. The primers used were synthesized by Sangon (Shanghai, China), and the sequences were as follows (5’–3’): CHCHD2, Forward: TACCAGGAGCCTCAGGGAAC, Reverse: CAAGTCGGCACTGTTTCAGC; TRIM27, Forward: GTACTTCGCAGAGCCCATGA, Reverse: TACCAGTTGGGTCACGTTGG; ABCC1, Forward: ATCACCTTCTCCATCCCCGA, Reverse: TTCTGAATCCAGGCCTGCTG; CASP10, Forward: ATCCTTTCGGCATGTGGAGG, Reverse: GGCTGGGGCATCTGTTTCTT; IFITM3, Forward: CACTGTCCAAACCTTCTTCTCTC, Reverse: TCACGTCGCCAACCATCTTC. The concentration of the primers used for RT-PCR was 400 nM.

## Supplementary Materials

The following are available online. Table S1: HSV-1/mock ratios of quantified proteins in HSV-1-infected HEK 293T cells.
